# Prevalence and risk factors contributing to antibiotic-resistant *Staphylococcus aureus* isolates from poultry meat products in South Africa, 2015–2016

**DOI:** 10.4102/jsava.v90i0.1738

**Published:** 2019-08-29

**Authors:** Vashnee Govender, Evelyn Madoroba, Kudakwashe Magwedere, Geoffrey Fosgate, Lazarus Kuonza

**Affiliations:** 1 School of Health Systems and Public Health, Faculty of Health Sciences, University of Pretoria, Pretoria, South Africa; 2 South African Field Epidemiology Training Programme, National Institute for Communicable Diseases, Sandringham Johannesburg, South Africa; 3 Department of Agriculture, Forestry and Fisheries, Directorate of Veterinary Public Health, Pretoria, South Africa; 4 Onderstepoort Veterinary Institute, Feed and Food Analysis Laboratory, Agricultural Research Council, Onderstepoort, Pretoria, South Africa; 5 Department of Production Animal Studies, Faculty of Veterinary Science, University of Pretoria, Onderstepoort, Pretoria, South Africa

**Keywords:** *Staphylococcus aureus*, antimicrobial resistance, poultry, meat safety, MRSA, methicillin-resistant *S. aureus*

## Abstract

*Staphylococcus aureus*, including methicillin-resistant strains, has been detected in food products of animal origin globally. Limited data have been reported on the factors contributing to antibiotic resistance of food-borne pathogens in South Africa. The primary aim of this study was to determine the prevalence of *S. aureus*, including antibiotic-resistant strains, in poultry meat products as well as the evaluation of potential risk factors for contamination of poultry meat products with antibiotic-resistant *S. aureus* isolates. A cross-sectional investigation was conducted in municipalities located across the nine provinces of South Africa, which included abattoirs, meat processing facilities, retail outlets and cold stores at the major ports of entry into South Africa. *Staphylococcus aureus* isolates obtained from various poultry meat products were tested for susceptibility to 14 antibiotic compounds representing 10 antibiotic classes using the Kirby–Bauer disc diffusion method. Potential risk factors were evaluated using a logistic regression model. Of the 311 samples tested, 34.1% (*n* = 106) were positive for *S. aureus* (95% confidence interval [CI], 28.9% – 39.7%). Seventy-two of the 106 isolates were randomly selected for antibiotic sensitivity testing. Twenty-one per cent (*n* = 15) of the isolates selected for sensitivity testing were methicillin-resistant strains (95% CI, 12.2% – 32.0%). Multi-drug resistance was detected in 22.2% (*n* = 16) of these isolates tested (95% CI, 13.3% – 33.6%). Origin of the product (*p* = 0.160), type of meat product (*p* = 0.962), type of facility (*p* = 0.115) and facility hygiene practices (*p* = 0.484) were not significantly associated with contamination of poultry meat products with methicillin-resistant strains. The study provides baseline data for further studies on antibiotic resistance risk assessments for food-borne pathogens, including *S. aureus*, which should guide the implementation plans of the South African National Antimicrobial Resistance Strategy Framework, 2017–2024.

## Introduction

*Staphylococcus aureus* is a human and animal commensal bacterium with the potential to cause disease in susceptible hosts (Gosbell & Van Hal 2013). It produces enterotoxins, which accumulate in food items during production and processing and which may cause food poisoning (Centers for Disease Control and Prevention 2011; Gosbell & Van Hal 2013; Grumann, Nübel & Bröker 2014). Although the bacteria may be destroyed by heating contaminated food items, their toxins are heat resistant (Centers for Disease Control and Prevention 2011; Grumann et al. 2014).

It is estimated that 25% of healthy people are carriers of *S. aureus* (Centers for Disease Control and Prevention 2011). Infection manifests clinically as dermatitis and life-threatening systemic infections (Naber 2009; World Health Organization [WHO] 2016). These infections are generally considered to be susceptible to antibiotic chemotherapy, but with increased resistance to available antibiotics. This has contributed to the evolving epidemiology of methicillin-resistant *S. aureus* (MRSA) (Naber 2009). Hospital-acquired MRSA (HA-MRSA) is viewed as one of the first types of multi-resistant nosocomial pathogens. New lineages of MRSA have emerged that cause infection in people without contact with healthcare systems and are referred to as community-associated MRSA (CA-MRSA) (Pantosti & Venditti 2009). Cattle, pigs and poultry are also known to be asymptomatic carriers of livestock-associated MRSA (LA-MRSA) (Cuny, Wieler & Witte 2015).

Antimicrobial resistance (AMR) presents a major threat to global public health and has become a serious challenge in clinical practice and healthcare (Laxminarayan et al. 2013; McAdam et al. 2012; WHO 2016). Because of resistance to existing antibiotics and the lack of development of new antibiotic compounds, higher costs and reduced efficacy of treatment for common infections are experienced in healthcare settings (Centers for Disease Control and Prevention 2011; Laxminarayan et al. 2013; McAdam et al. 2012; WHO 2016).

Previously, MRSA strains were considered to be acquired exclusively through nosocomial transmission (Naber 2009). Methicillin-resistant *S. aureus* strains are present in communities and have also been detected in a variety of food products of animal origin in countries throughout the world (European Food Safety Authority 2009; Jones et al. 2002; Kluytmans 2010; Newell et al. 2010). The possibility of people acquiring antibiotic-resistant *S. aureus* infections through contaminated food products has been reported (European Food Safety Authority 2009; Jones et al. 2002; Kluytmans 2010; Newell et al. 2010). The ST398 MRSA strain has been identified in pig, cattle and poultry production systems and zoonotic transmission has been recognised (European Food Safety Authority 2009). The Dutch Food Safety Agency reported that 85% of the 264 MRSA isolates from 2217 retail meat samples were the ST398 strain (European Food Safety Authority 2009). The risk of food poisoning because of *S. aureus* in food products is not particularly dependent on whether resistant genes are present or not and the development of disease through the ingestion of contaminated food is rare but possible under extreme patient conditions (European Food Safety Authority 2009). However, the risk for colonisation during handling of contaminated products is more relevant and is dependent on various environmental, host and pathogen factors (European Food Safety Authority 2009). Ho, O’Donoghue and Boost (2013) reported that the handling of raw meat posed an occupational risk to food handlers for nasal colonisation by *S. aureus*. Heysell et al. (2011) reported that 21% of tuberculosis patients in a rural KwaZulu-Natal hospital were found to be nasal carriers of MRSA and 90% of these cultures were found in patients with concurrent HIV infection.

The aim of this study was to determine the prevalence of *S. aureus*, including antibiotic-resistant strains, in poultry meat products and to identify and evaluate potential risk factors that could contribute to the contamination of poultry meat products with antibiotic-resistant *S. aureus* isolates in South Africa. We investigated the hypothesis that specific factors related to the origin of meat products, type of meat products, type of facility and facility hygiene contribute to the contamination of poultry meat products with antibiotic-resistant *S. aureus*. The findings of the study could assist food safety policy-makers in identifying possible policy gaps and areas of focus for strengthening meat safety, thereby reducing the risk of disease exposure to susceptible people.

## Materials and methods

Data on registered Food Business Operators (FBOs) were collected from environmental health officers of each municipality throughout South Africa (Table 1) through personal structured interviews conducted by the Department of Agriculture, Forestry and Fisheries (DAFF). Food Business Operators (i.e. abattoirs, meat processing facilities, butcheries, retail outlets and cold stores at the major ports of entry) were the sampling units. Samples were collected from January 2015 to August 2016.

**TABLE 1:** Origin and number of poultry meat samples collected from 2015 to 2016.

Province (*n*)	Municipality	No. of samples
Western Cape (39 = 12.5%)	West Coast District City of Cape Town Metropolitan	1 38
Northern Cape (12 = 3.9%)	Pixley ka Seme District ZF Mgcawu District Frances Baard District John Taolo Gaetsewe District	1 4 4 3
Eastern Cape (64 = 20.6%)	Buffalo City Metropolitan Nelson Mandela Bay Metropolitan	8 56
Free State (26 = 8.4%)	Lejweleputswa District Thabo Mofutsanyana District Fezile Dabi District Mangaung Metropolitan	3 5 8 10
KwaZulu-Natal (37 = 11.9%)	uMgungundlovu District Amajuba District eThekwini Metropolitan	6 1 30
Mpumalanga (52 = 16.7%)	Gert Sibande District Nkangala District Ehlanzeni District	18 18 16
Limpopo (7 = 2.3%)	Capricorn District Waterberg District	5 2
North West (57 = 18.3%)	Bojanala Platinum District Ngaka Modiri Molema District Dr Ruth Segomotsi Mompati District Dr Kenneth Kaunda District	9 22 11 15
Gauteng (17 = 5.5%)	West Rand District Ekurhuleni Metropolitan City of Johannesburg Metropolitan City of Tshwane Metropolitan	3 5 7 2

No., number.

A cross-sectional study was conducted to determine the prevalence of antibiotic resistance among *S. aureus* isolates and to identify potential risk factors for contamination of poultry meat samples. Samples from various municipalities situated in all nine provinces and the major ports of entry to South Africa were included in this study from January 2015 to August 2016. The municipalities within the provinces that responded to the request from DAFF to participate in the project were included in the sampling frame. Considering the nature of this study, sampling all the FBOs registered by each responding municipality was impractical and therefore a sample was drawn to best represent the total FBOs in South Africa using non-probability sampling.

The number of registered abattoirs in all nine provinces was estimated at 612. High throughput and low throughput abattoirs were targeted in the study with the exclusion of non-slaughtering and infrequently slaughtering abattoirs. Fifty-two municipalities reported the registration of 485 FBOs producing ready-to-eat (RTE) meat products, while municipalities with no registered RTE FBOs and abattoirs were excluded. Selection of FBOs within a municipality was targeted, based on confirmation of production activities on the day of sampling. At each FBO, composite samples were extracted from five individual sampling points (Department of Agriculture, Forestry and Fisheries 2012). Samples were randomly collected by Veterinary Public Health Officers from the selected FBOs registered by co-operating municipalities in the respective provinces.

The facilities involved at the major ports of entry, that is, Durban, Cape Town and Port Elizabeth, were cold stores. The selected three ports of entry had less than 40 registered cold stores. The cold stores were randomly selected. At the cold store, products were stratified into red meat and poultry where consignments were randomly selected for sampling from each stratum. Within a consignment, cartons were randomly selected from between 2000 and 2700 cartons constituting a consignment per container for a composite sample from five randomly selected cartons.

The minimum sample size for this study was calculated (Dohoo, Martin & Stryhn 2009) as 385. Information pertaining to sample type and origin was captured on sample submission forms and DAFF VPH officers assessed the facility hygiene using a questionnaire with scoring criteria during sample collection. The information captured pertaining to the products sampled were the country of origin, full product description, other ingredients (for processed meat samples) and the storage temperature. The information captured pertaining to the facility where the product was sampled included location, registered description, units of output and hygiene. Aspects of hygiene evaluated included the quality of General Hygiene Principles delivery in practice, hygiene training of employees, status of maintenance of premises, status of workforce, status of management and status of prerequisite programmes documentation and records. The data captured from laboratory testing involved the detection of *S. aureus* (by culture, staphylase testing and microscopic examination) and antibiotic sensitivity testing results.

The ISO 6888-1:1999 methods for the detection and identification of *S. aureus* were adapted for this study (International Organization for Standardization 2015). Meat products were cut and weighed to obtain 25 g portions before 225 mL of buffered peptone water (Oxoid, Basingstoke, United Kingdom [UK]) was added to each sample in sterile sample bags. The samples were macerated with the use of a Stomacher (Stomacher Lab Blender 400, Seward Ltd., West Sussex, UK) for 2 minutes. Duplicate plates of Baird Parker medium (Oxoid, Basingstoke, UK) were inoculated with 1 mL of sample suspension using the surface spread method and incubated for 48 hours at 37 °C. Black colonies surrounded by opaque zones were tested for agglutination using the Staphylase Test Kit (Oxoid, Basingstoke, UK). The detection of *S. aureus* was confirmed if isolates were coagulase positive, Gram positive and coccoid in shape.

*Staphylococcus aureus* (*n* = 72) isolates were tested for susceptibility to antibiotics representing 10 antibiotic classes (i.e. penicillins, cephalosporins, tetracyclines, lincosamides, aminoglycosides, macrolides, sulphonamides, quinolones, amphenicols and glycopeptides). The Kirby–Bauer disc diffusion susceptibility test and McFarland Standard according to the 2012 Clinical and Laboratory Standards Institute (CLSI) performance standards for antimicrobial susceptibility testing were applied. Suspensions of the bacteria were made up with physiological saline and colonies cultured on nutrient agar media. The 0.5 McFarland Standard was confirmed by the use of spectrophotometry with an absorbance range of 0.08–0.12 at a wavelength of 625 nm (nanometers). The bacterial suspension was inoculated on Mueller-Hinton agar plates by uniform streaking with sterile cotton swabs.

Antibiotics selected were penicillin (10 IU), oxacillin (5 *µ*g), ampicillin (10 *µ*g), cefoxitin (30 *µ*g), vancomycin (30 *µ*g), erythromycin (30 *µ*g), gentamycin (10 *µ*g), clindamycin (2 *µ*g), sulphamethoxazole trimethoprim (25 *µ*g), florfenicol (30 *µ*g), ciprofloxacin (5 *µ*g), enrofloxacin (5 *µ*g), oxytetracycline (30 *µ*g) and ceftiofur (30 *µ*g). The antibiotic discs were applied using a disc dispenser onto commercially prepared Mueller-Hinton agar plates. A maximum of five antibiotic discs were applied per 90 mm plate. The plates were incubated at 35 °C and the zone diameters were measured after 18 h. The zone diameters were re-evaluated after 24 h of incubation at 35 °C for cefoxitin, oxacillin and vancomycin (CLSI 2012).

The *S. aureus* American Type Culture Collection strain 25 923 was used as a control for antibiogram testing. Antibiotic resistance was defined as *S. aureus* isolates demonstrating growth within the zone diameter interpretative standard for *S. aureus* species, as stated in the 2012 CLSI performance standards for antibiotic susceptibility testing. Isolates with intermediate sensitivity were classified as sensitive. Multi-drug-resistant isolates were classified as per the European Society of Clinical Microbiology and Infectious Diseases definition of non-susceptibility to at least one agent in three or more antibiotic classes (Magiorakos et al. 2012). Cefoxitin was used as a surrogate marker for the detection of MRSA (Fernandes, Fernandes & Collignon 2005). Oxacillin was included in the testing panel for comparative purposes.

The data from the sample submission forms and facility hygiene questionnaires were digitalised by double entry into Microsoft Excel 2010. These data were exported to STATA (Software for Statistics and Data Science) version 13.0 software programme for statistical analysis using descriptive and analytical methods.

The prevalence of antibiotic-resistant *S. aureus* was determined from proportional calculations.

The origin of the sample was classified as local or imported and was based on the origin of the animals. The product type was classified as unprocessed for anatomically recognisable meat cuts and processed for all other products. Retail and cold storage facilities were classified as storage facilities, while facilities involved with meat production and processing were classified as production facilities. Scores for facility hygiene practices below 50% were classified as poor, from 50% to 69% were classified as satisfactory and from 70% to 100% were classified as good.

### Ethical considerations

Ethics approval for the study was obtained from the University of Pretoria, Faculty of Health Sciences Research Ethics Committee. Written permission was obtained for the use of data from the DAFF project from the project owner, the Directorate: Veterinary Public Health. Anonymity of facilities will be maintained in the public domain. The findings of the study have been notified to the relevant regulatory authority for the investigation of potential legislative non-compliances.

## Results

The origin and number of poultry meat samples included in this study are presented in Table 1. The total number of poultry meat samples collected was 553, and 311 of the samples were included in this study (Table 1), of which 106 (34.1%) contained *S. aureus* (Table 2). Two hundred and forty-two samples and 32 *S. aureus* isolates were not tested because of resource constraints. Seventy of the 106 *S. aureus* isolates were found in local meat products (66.0%; 95% confidence interval [CI], 56.1% – 74.8%) and the remaining 36 isolates in imported products (34%; 95% CI, 25.2% – 43.9%). The vast majority of *S. aureus* isolates (98 out of 106) were contained in recognisable meat cuts (92.5%; 95% CI, 85.2% – 96.5%), with the remaining eight isolates detected in processed meat products (7.5%; 95% CI, 3.6% – 14.8%). With regard to the type of facility, 34.9% (95% CI, 26.1% – 44.9%) of isolates were found in products sampled from retail facilities and 33.9% (95% CI, 25.2% – 43.9%) from cold stores. The prevalence of *S. aureus* in products by facility hygiene assessment was determined to be 45.5% in products sourced from facilities with good hygiene scores (95% CI, 30.7% – 60.2%), 28.6% in products sourced from facilities with satisfactory hygiene scores (95% CI, 9.2% – 47.9%) and 44.4% in products sourced from facilities with poor hygiene scores (95% CI, 25.7% – 63.2%). The prevalence of *S. aureus* in products sourced from facilities where the hygiene was not assessed was 31.1% (95% CI, 24.9% – 37.2%).

**TABLE 2:** Prevalence of *Staphylococcus aureus* in poultry meat products, South Africa, 2015–2016.

Product origin and description		Poultry samples tested (*n* = 311)	Samples tested positive for *S. aureus* (*n* = 106)	Prevalence (%)	95% CI
Origin of product				
Local	197	70	35.5	28.9–42.2
Imported	114	36	31.6	23.0–40.1
Type of product				
Recognisable meat cuts	265	98	37.0	31.2–42.8
Processed raw	29	5	17.2	3.5–31.0
Processed ready-to-eat	12	3	25.0	0.5–49.5
Offal	5	0	0.0	-
Type of facility				
Cold store	114	36	31.6	23.0–40.1
Retail	103	37	35.9	26.7–45.2
Butchery	69	26	37.7	26.2–49.1
Abattoir	17	6	35.3	12.6–58.0
Processing plant	8	1	12.5	2.2–47.1
Facility hygiene				
Good	44	20	45.5	30.7–60.2
Satisfactory	21	6	28.6	9.2–47.9
Poor	27	12	44.4	25.7–63.2
Not assessed (missing)	219	68	31.1	24.9–37.2

CI, confidence interval; *S. aureus, Staphylococcus aureus.*

Antibiotic sensitivity testing was conducted on 72 out of 106 *S. aureus* isolates from the 311 samples (Tables 3 and 4).

**TABLE 3:** Prevalence of antibiotic resistance among *Staphylococcus aureus* isolates (*n* = 72) from poultry meat products in South Africa, 2015–2016.

Product origin and description	MRSA† isolates (*n* = 15)			Multi-drug-resistant isolates‡ (*n* = 16)		
	*n*	§Prev%	95% CI¶	*n*	§Prev%	95% CI¶
Origin of product						
Local	7	15.5	6.5–29.5	8	17.8	8.0–32.1
Imported	8	29.6	13.8–50.2	8	29.6	13.8–50.2
Type of product						
Recognisable meat cuts	14	20.9	11.9–32.6	15	22.4	13.1–34.2
Processed raw	1	25.0	0.6–80.6	1	25.0	0.6–80.6
Processed ready-to-eat	0	0	-	0	0.0	-
Offal	0	0	-	0	0.0	-
Type of facility						
Cold store	8	29.6	13.8–50.2	8	29.6	13.8–50.2
Retail	6	20.7	8.0–39.7	7	24.1	10.3–43.5
Butchery	1	7.1	0.2–33.9	1	7.1	0.2–33.9
Abattoir	0	0	-	0	0.0	-
Processing plant	0	0	-	0	0.0	-
Facility hygiene						
Good	2	14.3	1.8–42.8	2	14.3	1.8–42.8
Satisfactory	0	0	-	0	0.0	-
Poor	0	0	-	0	0.0	-
Not assessed (missing)	13	28.3	16.0–43.5	14	30.4	17.7–45.8

MRSA, methicillin-resistant *S. aureus.*

† , Methicillin-resistant *Staphylococcus aureus.*

‡ , *Staphylococcus aureus* isolates resistant to three or more groups of antibiotics.

§ , Prevalence among 72 *Staphylococcus aureus* isolates.

¶ , 95% confidence interval.

**TABLE 4:** Distribution of antibiotic-resisant *Staphylococcus aureus* isolates (*n* = 72) from poultry meat products in South Africa, 2015–2016.

Antibiotic class	Antibiotic	Resistant (%)		Intermediate sensitivity (%)		Susceptible (%)	
		*n*	%	*n*	%	*n*	%
Penicillins	Penicillin	20	27.8	-	-	52	72.2
	Ampicillin	16	22.2	-	-	56	77.8
	Oxacillin	11	15.3	4	5.6	57	79.2
Cephalosporins	Cefoxitin†	15	20.8	-	-	57	79.2
	Ceftiofur	4	5.6	6	8.3	62	86.1
Tetracyclines	Oxytetracycline	31	43.1	1	1.4	40	55.6
Lincosamides	Clindamycin	16	22.2	10	13.9	46	63.9
Aminoglycosides	Gentamicin	13	18.1	-	-	59	81.9
Macrolides	Erythromycin	10	13.9	9	12.5	53	73.6
Sulphonamides	Sulphamethoxazole	5	6.9	-	-	67	93.1
Quinolones	Enrofloxacin	1	1.4	6	8.3	65	90.3
	Ciprofloxacin	0	-	5	6.9	67	93.1
Glycopeptides	Vancomycin	0	-	-	-	72	100.0
Amphenicols	Florfenicol	0	-	-	-	72	100.0

† , Used as a surrogate marker for methicillin.

The prevalence of antibiotic-resistant *S. aureus* isolates among the 72 *S. aureus* isolates tested was 55.6% (*n* = 40, 95% CI, 43.4% – 67.3%). The prevalence of MRSA, as determined by resistance to cefoxitin, was 20.8% (95% CI, 12.2% – 32.0%), as observed in 15 out of 72 (Table 1). Multi-drug resistance (resistance to three and more antibiotic classes, including penicillins) was observed in 16 of 72 *S. aureus* isolates (22.2%, 95% CI, 13.3% – 33.6%).

Thirty-two (44.4%) isolates (95% CI, 32.7% – 56.6%) were susceptible to all 14 antibiotics (10 antibiotic classes). None of the 72 isolates tested were resistant to vancomycin, florfenicol or ciprofloxacin (Tables 3 and 4). More *S. aureus* isolates demonstrated resistance to penicillin and oxytetracycline than to any of the other antibiotics included in the sensitivity testing panel. Forty-three per cent (31 of 72) of *S. aureus* isolates (95% CI, 31.4% – 55.3%) demonstrated resistance to oxytetracycline (Table 4).

**FIGURE 1:**
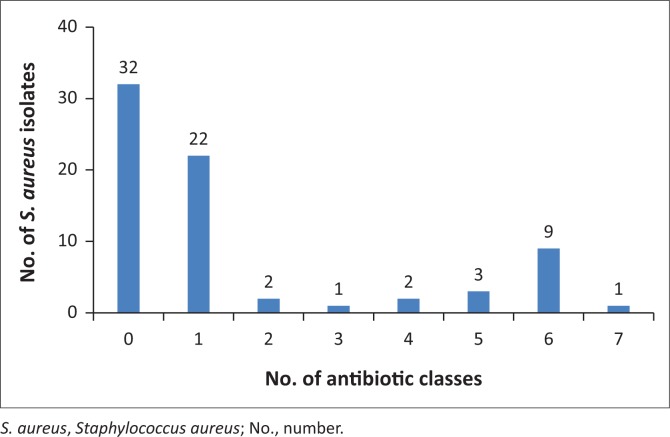
Distribution of antibiotic-resistant *Staphylococcus aureus* isolates (*n* = 72) from poultry meat samples in South Africa, 2015–2016.

Forty of the 72 isolates (55.6%; 95% CI, 44.1% – 67.0%) demonstrated resistance to at least one of the antibiotics tested. Twenty-four of the 72 *S. aureus* isolates were resistant to one and two antibiotic classes (33.3%; 95% CI, 22.7% – 45.4%) (Figure 1). Nine isolates (12.5%; 95% CI, 4.9% – 24.1%) exhibited resistance to six different antibiotic classes and one isolate (1.4%; 95% CI, 0.0% – 0.1%) was resistant to nine antibiotics (cefoxotin, oxacillin, penicillin, ampicillin, oxytetracycline, sulphamethoxazole, clindamycin, gentamicin and erythromycin) representing seven antibiotic classes.

## Discussion

The 14 antibiotics were selected on the basis of use in animal production systems (Andreasen 2013; Eagar, Swan & Van Vuuren 2012) and treatment of infections caused by bacteria in humans (Auwaerter 2016). Although only 72 out of the 106 isolates were tested for antibiotic sensitivity, the distribution of the 72 isolates in terms of product origin and description was similar to that of the 106 isolates because the 72 isolates were randomly selected for sensitivity testing. From this study, the prevalence of *S. aureus* in poultry meat samples on the South African market between 2015 and 2016 was estimated to be 34.1%. This is below the estimation of 44% made by Oguttu et al. (2014) for RTE chicken in Tshwane, Gauteng Province, South Africa. This variation in observation might be because of the sourcing of the RTE chicken products from an informal market where regulated hygiene measures are not likely to be implemented and enforced.

The reports of prevalence of *S. aureus* in poultry meat products internationally vary considerably: 6.42% in Iran (Madahi et al. 2014), 18.18% in Thailand (Akbar & Anal 2013), 43.3% in Turkey (Gundogan et al. 2005) and 28.6 % in Italy (Pesavento et al. 2007). In the United States, reports varied between 25.0% in Michigan (Bhargava et al. 2011), 17.8% in Iowa (Hansona et al. 2011), 42.1% in Oklahoma (Abdalrahman et al. 2015) and up to 41% in a collective study of five major US cities (Waters et al. 2011).

The prevalence of MRSA among *S. aureus* isolates from poultry meat products considered in this study was 20.8%. International studies reported this prevalence to be 7.89% in Thailand (Akbar & Anal 2013), 37.2% in Germany (Feßler et al. 2011), 1.6% in Italy (Normanno et al. 2007), 24.8% in the Netherlands (De Boer et al. 2009) and 26% in a collective study of five major US cities (Waters et al. 2011). The variation observed might be attributed to differences in the regulation of antibiotics used and hygiene management in production systems that were included in these studies.

**TABLE 5:** Univariate logistic regression results for methicillin-resistant *Staphylococcus aureus* detection as the outcome.

Risk factors for the outcome – MRSA detection		No. of MRSA isolates	Total *S. aureus* (*n* = 72)	Univariate odds ratio	95% CI	*p*
Origin of sample	Local†	7	45	2.29	0.72–7.2	0.160
	Imported	8	27	-	-	-
Type of product	Processed†	1	5	1.06	0.11–10.22	0.962
	Unprocessed	14	67	-	-	-
Type of facility	Processing†	1	16	5.46	0.66–45.04	0.115
	Storage	14	56	-	-	-
Facility hygiene	Poor†	0	9	0.33	0.14–7.55	0.484
	*Satisfactory*	2	17	-	-	-

CI, Confidence interval; MRSA, methicillin-resistant *S. aureus*.

† , Reference category per variable.

Multi-drug resistance was observed in 22.2% of isolates. The highest resistance of *S. aureus* isolates observed was against oxytetracycline (43.1%) and penicillin (23.8%). Resistance to oxytetracycline was also reported to be the highest among antibiotics tested in Thailand (Akbar & Anal 2013). *Staphylococcus aureus* is well known to express the highest resistance to penicillin among the beta-lactam antibiotic class, and penicillin resistance by Gram positive bacteria has been reported since 1940 (Laxminarayan et al. 2013). The resistance to oxytetracycline is not surprising because this is one of the most commonly used antibiotics in South African livestock production systems. Oxytetracycline is registered for use in animal feed under the *Fertilizers, Farm Feeds and Agricultural Remedies Act* (Act No. 36 of 1947) and tetracyclines were reported to be the second most consumed class of antibiotic by the South African animal production industry (Eagar et al. 2012).

Previous studies have linked the detection of *S. aureus* and MRSA isolates in meat products to food handlers and poor hygiene practices during production processes. However, this study failed to demonstrate significant associations between the presence of MRSA in poultry meat products and facility factors, including processing and hygiene practices. This finding could indicate that contamination of products with the bacteria was not associated with slaughter and processing, but rather that the birds already carried the bacteria before presentation for slaughter and that the bacteria are most likely acquired either during rearing on farms or during handling before transport and slaughter. This inference is plausible because chickens, as well as other livestock species such as pigs and cattle, are known to be carriers of *S. aureus*, which is supported by the study conducted by Mkhize, Zishiri and Mukaratirwa (2017), which found that faecal and caecal samples tested from commercial broiler birds in KwaZulu-Natal, South Africa, contained antibiotic-resistant *S. aureus* bacteria. However, the lack of statistically significant findings could also be because of the study lacking sufficient power to detect significant associations as a result of the inadequate sample size.

This study had several limitations. With regard to the sampling, the number of samples analysed per province was not proportional to production or consumption in each province. Samples may have been misclassified by country of origin because packaging at retail outlets does not always carry the original labelling from the country of origin. All 553 samples and 106 *S. aureus* isolates could not be tested for antibiotic sensitivity because of limitations of laboratory resources and time constraints, which could have introduced sample bias; however, a comparison of the distribution of the 553 samples collected with those 311 included in the study (Table 1) did not reflect major differences between the groups. Convenience sampling of municipalities and establishments was conducted and therefore not necessarily representative of the entire country. The data were incomplete for facility hygiene, sample temperatures and enumeration of *S. aureus* from samples. In general, risk factors relating to animal health and environmental factors such as feed and farm hygiene were not accounted for in this study.

Despite the limitations of the study, the findings nevertheless present pertinent questions, including the effects of exposure to antimicrobial-resistant organisms from products of animal origin on people and how prevention of exposure to antimicrobial-resistant organisms in products of animal origin can be achieved. The association between the presence of antibiotic-resistant organisms in products of animal origin and infections with these organisms is not clear. This must prompt further studies on such a relationship, particularly where these organisms were thought to be exclusively encountered through nosocomial routes.

The findings of this study highlight the need to identify the source of antibiotic-resistant pathogens in food products of animal origin. This will support the identification of existing policy gaps and weaknesses. The resolution on AMR, adopted at the 39th Food and Agriculture Organization of the United Nations (FAO) conference in June 2016, urged member countries to develop or strengthen national plans, strategies and international collaboration for the surveillance, monitoring and containment of AMR in food, agriculture and the environment, in close coordination with related plans for human health. The management of AMR and food safety must be tackled using the One Health approach, as advocated by the FAO, World Organisation for Animal Health (OIE) (2010) and WHO.
